# Pharmacogenetic profiling and cetuximab outcome in patients with advanced colorectal cancer

**DOI:** 10.1186/1471-2407-11-496

**Published:** 2011-11-25

**Authors:** Laetitia Dahan, Emmanuelle Norguet, Marie-Christine Etienne-Grimaldi, Jean-Louis Formento, Mohamed Gasmi, Isabelle Nanni, Jean Gaudart, Stéphane Garcia, L'Houcine Ouafik, Jean-François Seitz, Gérard Milano

**Affiliations:** 1 Assistance Publique-Hôpitaux de Marseille, Hôpital Timone, Université de la Méditerranée, Marseille, France; 2 Laboratoire d'oncopharmacologie, Centre Antoine Lacassagne, Nice, France; 3 Assistance Publique-Hôpitaux de Marseille, Hôpital Nord, Université de la Méditerranée, Marseille, France

**Keywords:** EGFR inhibitors, cetuximab, clinical outcome, colorectal cancer, polymorphisms

## Abstract

**Background:**

We analyzed the influence of 8 germinal polymorphisms of candidate genes potentially related to EGFR signalling (*EGFR*, *EGF*, *CCND1*) or antibody-directed cell cytotoxicity (*FCGR2A *and *FCGR3A*) on outcome of colorectal cancer (CRC) patients receiving cetuximab-based therapy.

**Methods:**

Fifty-eight advanced CRC patients treated with cetuximab-irinotecan salvage therapy between 2001 and 2007 were analyzed (mean age 60; 50 PS 0-1). The following polymorphisms were analyzed on blood DNA: *EGFR *(CA repeats in intron 1, -216 G > T, -191C > A, R497K), *EGF *(A61G), *CCND1 *(A870G), *FCGR2A *(R131H), *FCGR3A *(F158V). Statistical analyses were conducted on the total population and on patients with wt KRas tumors. All SNPs were considered as ternary variables (wt/wt *vs *wt/mut *vs *mut/mut), with the exception of -191C > A *EGFR *polymorphism (AA patient merged with CA patients).

**Results:**

Analysis of skin toxicity as a function of EGFR intron 1 polymorphism showed a tendency for higher toxicity in patients with a low number of CA-repeats (p = 0.058). *CCND1 *A870G polymorphism was significantly related to clinical response, both in the entire population and in KRas wt patients, with the G allele being associated with a lack of response. In wt KRas patients, time to progression (TTP) was significantly related to *EGFR *-191C > A polymorphism with a longer TTP in CC patients as compared to others, and to *CCND1 *A870G polymorphism with the G allele being associated with a shorter TTP; a multivariate analysis including these two polymorphisms only retained *CCND1 *polymorphism. Overall survival was significantly related to *CCND1 *polymorphism with a shorter survival in patients bearing the G allele, and to *FCGR3A *F158V polymorphism with a shorter survival in VV patients (in the entire population and in KRas wt patients). *FCGR3A *F158V and *CCND1 *A870G polymorphisms were significant independent predictors of overall survival.

**Conclusions:**

Present original data obtained in wt KRas patients corresponding to the current cetuximab-treated population clearly suggest that *CCND1 *A870G polymorphism may be used as an additional marker for predicting cetuximab efficacy, TTP and overall survival. In addition, *FCGR3A *F158V polymorphism was a significant independent predictor of overall survival.

## Introduction

Despite the introduction of new treatments, the 5-year survival rate for metastatic colorectal cancer (mCRC) remains below 10% [1]. Cetuximab, an IgG1 monoclonal antibody (MoAb) targeting epidermal growth factor receptor (EGFR), has proven to be effective in providing clinical benefit in approximately 10% to 20% of patients [2-4]. EGFR is a transmembrane tyrosine kinase receptor that, following ligand binding, triggers two main signaling pathways: the RAS-RAF-MAPK pathway which is involved in cell proliferation, and the PI3K-PTEN-AKT pathway which controls cell survival and motility [5].

While the presence of a *KRAS *mutation permits identification of tumors that are insensitive to these treatments, only less than half of patients with a *KRAS *wild type (wt) tumor will benefit from treatments, suggesting a role for additional mechanisms of resistance [6-10]. It thus appears necessary to better define the subpopulation of patients who truly benefit from cetuximab. One approach to resolving this question may be the application of pharmacogenetics, as recently reviewed by Coate and co-workers [11]. Yet, gene polymorphisms may affect pharmacodynamics of anti-EGFR therapies such as cetuximab, by introducing inter-patient variability at the level of the EGFR target itself, the EGF ligand, as well as in the immunological mechanism called antibody-dependent cellular cytotoxicity (ADCC).

Four functional *EGFR *variants have been associated with *EGFR *regulation [12-14]: a (CA)n repeat polymorphism in *EGFR *intron 1, a G > A single nucleotide polymorphism (SNP) at codon 497, and two SNPs -216 G > T and -191C > A located in the promoter region. Modulation of the EGFR ligand EGF and of the downstream EGFR signaling, including the cyclin-D1 gene (*CCND1*), may also play a role in modulating cetuximab activity. Functional variants have been described in the *EGF *5'-untranslated region (*EGF *61 G > A) [15,16], and in the exon 4 of the *CCND1 *gene (870A > G) [17,18]. The ADCC, mediated through Fc receptors (FcγR) carried by immune cells such as macrophages and natural killer cells, plays an important role in the antitumor effect of IgG1 antibodies, such as cetuximab [19,20]. The effectiveness of ADCC may depend on the degree of activation of FcγR and constitutional polymorphisms have been demonstrated on genes encoding for these receptors: a histidine (H)/arginine (R) polymorphism at position 131 for *FCGR2A *and a valine (V)/phenylalanine (F) polymorphism at position 158 for *FCGR3A *[21].

In the present study, we investigated possible associations between these genetic variants and clinical outcomes of advanced CRC patients treated with cetuximab. Clinical end points were skin toxicity, clinical response, time to progression (TTP) and overall survival (OS).

## Materials and methods

### Patients

Fifty-eight patients with advanced colorectal carcinoma were included in this retrospective pharmacogenetic study. All were treated between December 2001 and November 2007. Forty-four patients were treated at the Hôpital La Timone and 14 at the Hôpital Nord (Marseille). The study was carried out with ethics committee approval and patients signed a specific informed consent for pharmacogenetic analyses. Patient characteristics are shown in Table 1. Formalin-fixed, paraffin-embedded tumor material was collected retrospectively for 50 patients. After histological control (HES) and macro-dissection to select tumor areas containing at least 50% tumor cells, DNA was extracted, and activating mutations of *KRAS *gene at codon 12 and codon 13 were analyzed by direct sequencing (Big Dye Terminator cycle sequencing kit, Applied Biosystems, Foster City, CA) on a 3130 genetic analyzer (Applied Biosystems). A *KRAS *mutation was detected in 32% of patients (16/50). The majority of patients (55%) received cetuximab as third-line therapy, and the associated chemotherapy was irinotecan for 71% of patients. Thirteen patients out of 58 had received previous therapy with bevacizumab for their metastatic disease. Cetuximab was administered i.v. over 2 hr at day1-day8-day15 (400 mg/m^2 ^starting dose, 250 mg/m^2 ^for subsequent doses, 21-day cycles). Treatment was administered until disease progression or unacceptable toxicity.

**Table 1:** Patient characteristics (N = 58)

**Age **(years)	Mean	60.2
	Range	32-83
		
**Gender**	Men	36
	Women	22
		
**PS**	0	28
	1	22
	2	6
	3	2
		
**Adjuvant chemotherapy**	No	41
	Yes	17
		
**Primary tumor localization**	Right colon	6
	Left colon	30
	Rectum	21
	Unknown	1
		
**Metastasis characteristics**	Single	23
	Multiple	35
	Synchronous	36
	Metachronous	22
		
***KRAS *mutation status**	Non-mutated	34
	Mutated at codon 12 or 13	16
	Unknown	8
		
**Previous administration of bevacizumab for metastatic disease**	No	45
	Yes	13
		
**Line of cetuximab treatment**	First	1
	Second	8
	Third	32
	≥ 4^th^	17
		
**Chemotherapy associated with cetuximab**	None	2
	Irinotecan	41
	FOLFIRI	14
	FOLFOX	1
		
**Number of cetuximab cycles**	Mean	7.6
	Median	6.0
	Range	1-29

### Toxicity evaluation

Toxicity evaluation focused on cetuximab-related toxicity, i.e. acneiform rash. The maximum observed toxicity grade was recorded for all patients (N = 58), according to NCI-CTCAE v3.0.

### Efficacy evaluation

Best clinical response, assessed according to modified RECIST criteria, was assessable on 56 patients (2 patients were not evaluated because of early treatment interruption due to toxicity). Time to progression (TTP) and specific survival (cancer-related death) were computed from day-1 of cetuximab treatment. At time of analysis, 51 patients out of 56 assessable patients had progressed. Survival was recorded in 57 patients among whom 44 had died from their cancer and one had died from an independent cause. Median follow-up was 39.2 months (reverse Kaplan-Meier method).

### Pharmacogenetic analyses

Germinal polymorphisms of *EGFR, EGF, CCND1, FCGR2A *and *FCGR3A *genes, potentially linked to cetuximab pharmacodynamics, were analyzed on DNA extracted from a 9 ml blood sample (Paxgene Blood DNA kit, Prenalytics). With the exception of the *EGFR *intron-1 polymorphism, all other variants were investigated using a polymerase chain reaction-restriction fragment length polymorphism (PCR-RFLP) method (Table 2). Electrophoresis separation was performed on a 3% agarose gel. The CA-repeats polymorphism in intron 1 of *EGFR *gene was analyzed by fluorescent genotyping on CEQ-8000 Beckman-Coulter, as previously described [22]. Due to the large number of genotypes (between 14 and 20 CA repeats), patients were split into 2 groups (patients with the sum of CA repeats ≤ 35 *vs *others). Wild-type and mutated cell lines were used as controls.

**Table 2:** Characteristics of the PCR-RFLP methods used

Gene	SNP	Restriction Enzyme	Primers
*EGFR*	497R > K rs2227983	BstNI	5-TGCTGTGACCCACTCTGTCT-3 5-CCAGAAGGTTGCACTTGTCC-3
*EGFR*	-216 G > T rs712829	BseRI	5-CCACCGCCTCCGGCGGCCGCTGGCCTTG-3 5-CGGCGAGACACGCCCTTACCTTT-3
*EGFR*	-191C > A rs712830	SacII	5-CCACCGCCTCCGGCGGCCGCTGGCCTTG-3 5-CGGCGAGACACGCCCTTACCTTT-3
*EGF*	61A > G rs4444903	AluI	5-TGTCACTAAAGGAAAGGAGGT-3 5-TTCACAGAGTTTAACAGCCC-3
*CCND1*	870A > G rs603965	ScrFI	5-GTGAAGTTCATTTCCAATCCGC-3 5-GGGACATCACCCTCACTTAC-3
*FCGR2A*	131R > H rs1801274	BstUI	5-GGAAAATCCCAGAAATTCTCGC-3 5-CAACAGCCTGACTACCTATTACGCGGG-3
*FCGR3A*	158F > V rs396991	NlaIII	First PCR: 5-ATATTTACAGAATGGCACAGG-3 5-GACTTGGTACCCAGGTTGAA-3
			Second PCR: 5-ATCAGATTCGATCCTACTTCTGCAGGGGGCAT-3 5-ACGTGCTGAGCTTGAGTGATGGTGATGTTCAC-3

### Statistics

The Exact p values for Hardy-Weinberg equilibrium were tested on http://innateimmunity.net/IIPGA2. All SNPs were considered as ternary categorical variables (wt/wt *vs *wt/mut *vs *mut/mut), with the exception of -191C > A *EGFR *polymorphism for which the only AA patient was merged with heterozygous patients. Non-parametric tests were performed for comparisons (Mann-Whitney or Kruskal-Wallis). Pearson chi-square tests were applied for categorical variables, including linkage disequilibrium analyses. A logistic model was applied for estimation of odds ratio (OR) associated with toxicity (1 = grade 2-3, 0 = grade 0-1) or response (1 = CR+PR, 0 = SD+PD). TTP and survival curves were plotted according to the Kaplan-Meier method. The influence of the various tested parameters on TTP and survival was assessed by means of Log Rank test, or Cox analysis (for continuous variables or multivariate analysis). For stepwise multivariate analyses, the probabilities for entry and removal were 0.05 and 0.10, respectively. Whatever the gene polymorphism and the clinical end-point, we firstly performed univariate analyses, and then included the significant genotypes (p ≤ 0.050 from univariate analyses) in a single multivariate analysis. In addition, for efficacy end-points (response, TTP and survival), we conducted additional univariate and multivariate analyses on the sub-population of KRas wt patients. The p value considered as statistically significant (p ≤ 0.05, two-sided test) was not corrected for multiple testing. Statistics were performed on SPSS software (v15.0).

## Results

### Description of gene polymorphisms

Table 3 depicts the frequency of analyzed genotypes. Regarding intron 1 *EGFR *polymorphism, homozygous 16-16 CA repeats was the most frequent genotype (29.3%), followed by heterozygous 16-20 CA repeats (22.4%) and 16-18 CA repeats (10.3%). All bi-allelic genotypes agreed well with those predicted by the Hardy-Weinberg equilibrium. No linkage disequilibrium was observed between *EGFR *-216 G > T, -191C > A and R497K polymorphisms, nor between *FCGR2A *H131R and *FCGR3A *V158F polymorphisms. Tumoral K-Ras mutation status was not related to polymorphisms of genes linked to the EGFR pathway (*EGFR, EGF, CCND1*).

**Table 3:** Distribution of gene polymorphisms

Gene	Genotype		N
***EGFR***	CA-repeats (intron 1)	Sum of CA ≤ 35	33
		Sum of CA > 35	23
	-216G>T	GG	16
		GT	29
		TT	11
	-191C>A	CC	45
		CA	11
		AA	1
	R497K	RR	29
		RK	18
		KK	8
***EGF***	61A>G	AA	25
		AG	21
		GG	9
***CCND1***	A870G	AA	9
		AG	35
		GG	12
***FCGR2A***	R131H	RR	20
		RH	29
		HH	7
***FCGR3A***	F158V	FF	30
		FV	20
		VV	6

### Impact of gene polymorphisms on toxicity

Maximum observed cetuximab-related acneiform rash was grade 0 in 7 patients, grade 1 in 14 patients, grade 2 in 28 patients and grade 3 in 9 patients, accounting for a total of 63.8% toxicity grade 2-3. This toxicity was not related to performance status or patient age, but was unexpectedly linked to patient gender, with a significantly greater toxicity in men as compared to women (77.8% *vs *40.9%, p = 0.005).

**Figure 1:**
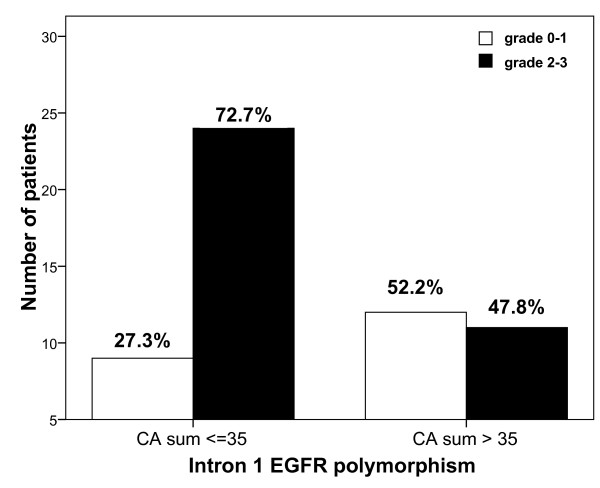
**Relationship between cetuximab-related acneiform rash (maximum observed grade) and CA-repeats polymorphism in intron 1 of *EGFR *gene (CA sum ≤ 35 *vs *CA sum > 35)**. Chi-square test: p = 0.058. OR relative to patients with CA sum > 35 was 2.91 (95% CI 0.95-8.92).

A tendency was observed for greater cutaneous toxicity in patients bearing short CA repeats in intron 1 of *EGFR *gene, with 72.7% grade 2-3 in patients with CA sum ≤ 35 *vs *47.8% in patients with CA sum > 35 (Figure 1, p = 0.058, OR = 2.91, 95% CI 0.95-8.92). No relevant relationship was observed for the other analyzed polymorphisms.

### Impact of gene polymorphisms on response

Best clinical response was CR in one patient, PR in 5 patients, stabilization in 11 patients and progression in 39 patients (i.e. 10.7% response rate, CR+PR). Even though response rate was greater in patients developing cutaneous toxicity (13.9% in patients with grade 2-3 *vs *5.0% in patients with grade 0-1), this difference did not reach statistical significance (p = 0.30). In other respects, response rate was 15.2% in patients with non-mutated KRas tumors *vs *0% in patients with KRas mutated tumors (p = 0.11).

**Figure 2:**
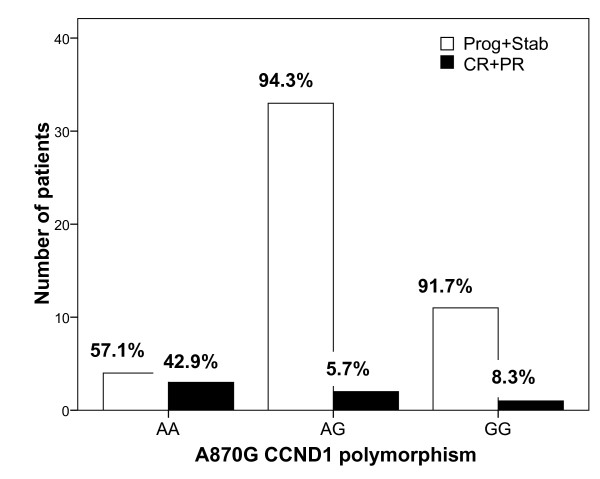
**Relationship between best clinical response and *CCND1 *A870G gene polymorphism on the whole population**. P value of chi-square test was 0.016 for AA *vs *AG *vs *GG, 0.004 for AA *vs *AG+GG and 0.73 for AA+AG *vs *GG. Response rate was 6.4% in AG+GG patients and 11.9% in AA+AG patients.

Regarding gene polymorphisms, *CCND1 *polymorphism at position A870G was significantly related to clinical response (Figure 2, AA *vs *AG *vs *GG, p = 0.016). Interestingly, an analysis restricted to patients with KRas wt tumors confirms the predictive value of *CCND1 *A870G polymorphism on clinical response (AA *vs *AG *vs *GG, p = 0.027), with the presence of the G allele being associated with a lack of response (AA *vs *AG+GG, p = 0.007, OR relative to GG+AG patients was 39.0, 95% CI 2.67-569.7). No relevant relationship was observed for the other analyzed polymorphisms.

### Impact of gene polymorphisms on TTP and specific survival

**Figure 3:**
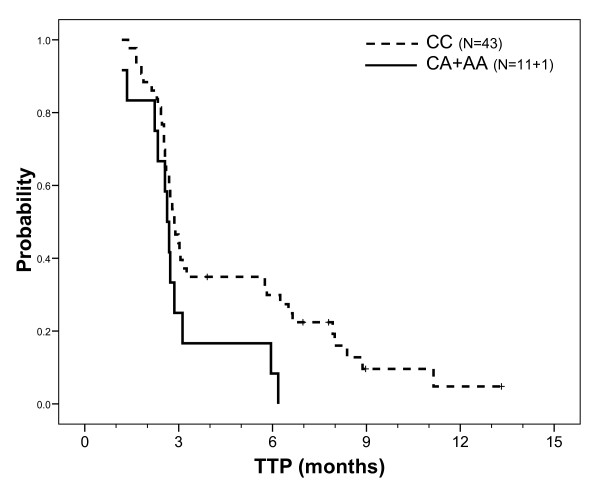
**TTP probability according to *EGFR *-191C > A gene polymorphism on the whole population**. Median TTP was 2.9 months in CC patients (43 patients, 38 events) *vs *2.6 months in CA+AA patients (12 patients, 12 events); Log Rank test: p = 0.050.

**Figure 4:**
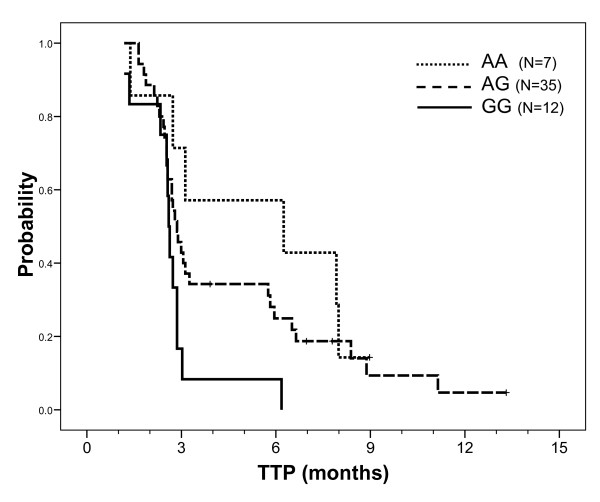
**TTP probability according to CCND1 A870G gene polymorphism on the whole population**. Median TTP was 6.3 months in AA patients (7 patients, 6 events) vs 2.9 months in AG patients (35 patients, 31 events) vs 2.6 months in GG patients (12 patients, 12 events); Log Rank test: p = 0.037. Comparison of AA+AG patients (median TTP 2.9 months) vs GG patients gave a p value at 0.016.

Median TTP was 2.8 months. TTP was not influenced by demographic or tumor characteristics, including K-Ras mutation status, with the exception of a slight influence of primary tumor localization (shorter TTP in patients with right colon primary cancer, p = 0.027). Univariate analyses showed that only -191C > A *EGFR *and A870G *CCND1 *genotypes were related to TTP. Figure 3 shows a trend for a longer TTP in homozygous *EGFR *-191CC patients relative to other patients (p = 0.050). *CCND1 *genotype had a significant impact on TTP, with a longer TTP in AA patients relative to GG patients and an intermediary TTP in heterozygous patients (p = 0.037, Figure 4). Kaplan-Meier analyses conducted in the sub-group of patients with KRas wt tumors reinforced the influence of both *EGFR *-191C > A (median 3.0 months in CC patients *vs *2.6 months in CA+AA patients p = 0.030) and *CCND1 *A870G (medians 7.9, 3.0 and 2.6 months in AA, AG and GG patients, respectively, p = 0.024) genotypes on TTP. A multivariate stepwise Cox analysis including both gene polymorphisms (considered as previously), on the whole population, only retained *CCND1 *polymorphism (p = 0.057). This latter statistic became significant (*CCND1 *p = 0.035, *EGFR *not retained in the analysis) in a multivariate stepwise analysis conducted on patients with KRas wt tumors (relative risk of progression in GG patients relative to AA patients was 5.59, 95% CI 1.36-22.95; relative risk of progression in AG patients relative to AA patients was 2.32, 95% CI 0.66-8.17).

**Figure 5:**
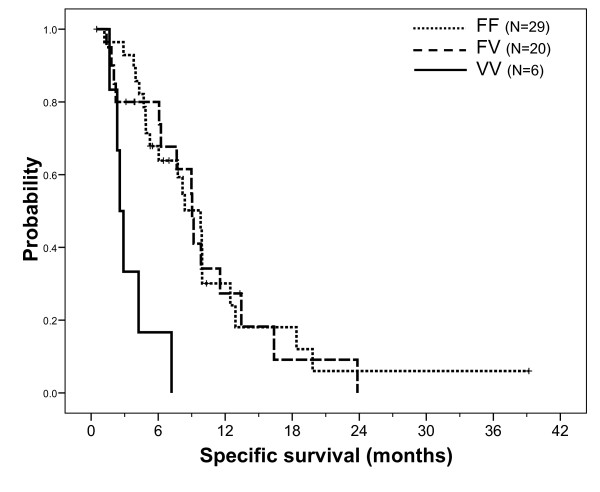
**Specific survival (cancer-related death) probability according to FCGR3A F158V gene polymorphism on the whole population**. Median survival was 9.8 months in FF patients (29 patients, 21 events) vs 9.0 months in FV patients (20 patients, 15 events) vs 2.6 months in VV patients (6 patients, 6 events); Log Rank test: p < 0.001. Comparison of FF patients vs others was not significant (p = 0.31) whereas comparison of VV patients vs others gave a p value < 0.001.

**Figure 6:**
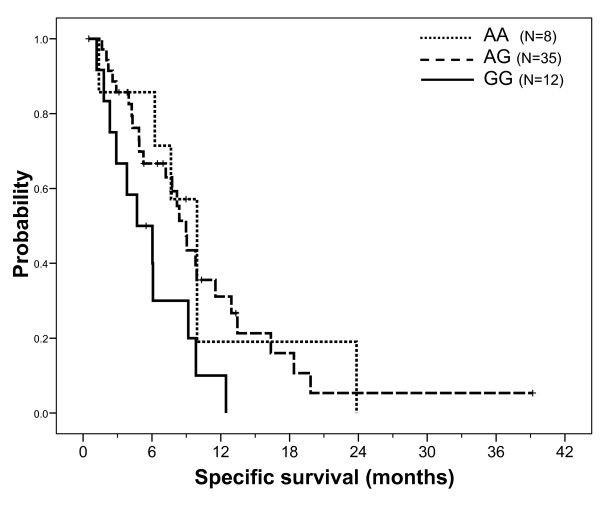
**Specific survival (cancer-related death) probability according to *CCND1 *A870G gene polymorphism in the whole population**. Median survival was 9.9 months in AA patients (8 patients, 6 events) *vs *9.0 months in AG patients (35 patients, 25 events) *vs *4.7 months in GG patients (12 patients, 11 events); Log Rank test: p = 0.050. Comparison of AA+AG patients (median survival 9.0 months) *vs *GG patients gave a p value at 0.015.

Median specific survival was 8.4 months. Specific survival was influenced by neither demographic nor tumor characteristics, including K-Ras mutation status. However, patients previously treated by bevacizumab had a significantly shorter survival (median 4.9 months, 13 patients, 11 cancer-related deaths) than those who did not receive bevacizumab (median 9.8 months, 44 patients, 33 cancer-related deaths, p = 0.018). Univariate analyses revealed a significant influence of *FCGR3A *F158V polymorphism on survival (FF *vs *FV *vs *VV, p < 0.001), with the 6 VV patients having a markedly shorter survival (Figure 5). The influence of *CCDN1 *A870G polymorphism was at the limit of significance (AA *vs *AG *vs *GG, p = 0.050, Figure 6), with GG patients exhibiting the poorest survival. Other gene polymorphisms had no influence on specific survival. Univariate analyses conducted in the sub-group of patients with KRas wt tumors confirmed the impact of *FCGR3A *F158V polymorphism on survival (median 9.9, 9.0 and 2.9 months in FF, FV and VV patients, respectively, p = 0.003) and reinforced the significance of *CCND1 *A870G polymorphism (medians 9.9, 9.9 and 2.9 months in AA, AG and GG patients, respectively, p = 0.024). A multivariate stepwise analysis conducted on the entire population, including both gene polymorphisms considered as ternary variables along with bevacizumab pre-treatment (yes/no), revealed that *CCND1 *A870G (p = 0.044) and *FCGR3A *F158V (p = 0.006) polymorphisms were significant independent survival predictors (p = 0.014 for bevacizumab pre-treatment). Finally, this latter result was confirmed in a multivariate stepwise analysis conducted in the sub-group of patients with wt KRas tumors (p values were 0.021, 0.036 and 0.058 for *CCND1*, *FCGR3A *and bevacizumab pre-treatment, respectively).

## Discussion

Cetuximab has shown efficacy in patients with metastatic colorectal cancer in several phase II trials leading, in 2004, to FDA approval for the treatment of irinotecan-refractory metastatic colorectal cancer. Several retrospective and prospective studies have clearly demonstrated that *KRAS *mutation confers resistance to these patients [6-10,23,24] but the complete mechanism of cetuximab sensitivity remains only partially understood. The present study was conducted in patients receiving cetuximab before KRas-mutation testing was introduced as a requirement. As expected, no response rate was observed in mutated *KRAS *patients *vs *15.2% in wt tumors, even though the difference did not reach significance. We presently analyzed 8 gene polymorphisms involving 5 relevant candidate genes potentially related to the pharmacodynamics of cetuximab, namely *EGFR*, *EGF*, *CCND1*, *FCGR2A *and *FCGR3A*, on 58 CRC patients receiving cetuximab-based therapy. Statistical analyses were conducted in the whole set of patients, as well as in the sub-group of 34 patients with wt KRas tumors, so as to reflect the current cetuximab-treated population.

Numerous studies have reported a relationship between favorable outcome of cetuximab-treated patients and related skin toxicity [2,3,25]. Accordingly, present data show a higher response rate in patients developing grade 2-3 cutaneous toxicity as compared to patients with grade 0-1 (14% *vs *5%, respectively), even though not significant. Present results also show a tendency for an association between intron 1 *EGFR *polymorphism and cetuximab-related skin toxicity: the incidence of grade 2-3 toxicity was 1.5-fold greater in patients bearing short CA-repeats in intron 1 of *EGFR *gene (CA sum ≤ 35) as compared to others (p = 0.058, Figure 1). This observation concords well with previous studies by Amador et al. [26] and Graziani et al. [27] reporting that patients developing cutaneous rash after anti-EGFR therapies presented shorter CA-repeats in intron 1 of *EGFR *gene as compared to patients who did not develop rash. Experimental studies have reported an inverse correlation between the number of CA-repeats in the intron 1 of the *EGFR *gene and *EGFR *gene transcription [28-30]. It can thus be hypothesized that elevated ubiquitous EGFR expression (including skin and tumor) renders the cells more susceptible to anti-EGFR effects.

In addition to the influence of intron 1 polymorphism on *EGFR *gene transcription, *EGFR *gene presents two functional polymorphisms in the promoter region: the -216G/T polymorphism located in a Sp1 binding site [31,32], and the -191C/A polymorphism located 4 bp upstream of a transcription initiation site [31]. These two SNPs may thus have an impact on *EGFR *gene regulation. Present data obtained on patients with wt KRas tumors show a significantly longer TTP in homozygous *EGFR *-191CC patients relative to other patients (p = 0.030, univariate analysis). However, this genotype was not retained in a multivariate analysis.

Cyclin D1 is a downstream effector of EGFR signaling that regulates cell cycle. The *CCND1 *A870G gene polymorphism affects the splice donor site at the exon 4/intron 4 boundary, resulting in two different mRNA transcripts (a and b) [33]. Both the A allele and the G allele can encode these two transcripts. However, the A allele preferentially encodes transcript b, which results in a longer half-life cyclin D1 protein [33]. The impact of *CCND1 *A870G polymorphism on cancer progression has been studied in head and neck cancer patients, with conflicting results [34,35]. In our study, patients homozygous for the *CCDN1 *870AA genotype had a significantly greater response rate than AG or GG patients, both in the whole population and in patients with a wt KRas tumor (75.0% *vs *7.1%, respectively in wt KRas patients). In addition, patients with the *CCND1 *870AA genotype had a significantly longer median TTP than GG patients, with AG patients having an intermediary TTP, both in the whole population and in patients with a wt KRas tumor (median TTP were 7.9, 3.0 and 2.6 months, in AA, AG and GG wt KRas patients, respectively). Of note, in patients with wt KRas tumors, *CCND1 *polymorphism also influenced specific survival, with a significantly shorter survival in GG patients. The positive influences of *CCND1 *870A allele are thus consistent with one another, even though they do not concord with the sole published study having analyzed the impact of *CCND1 *A870G polymorphism on the outcome of advanced colorectal cancer patients receiving cetuximab therapy [36]. In this latter study, conducted on a limited sample of 39 patients, the 870 G allele had a favorable impact on survival [36].

In addition to direct anti-EGFR effect, IgG1 mAbs such as cetuximab mediate anti-tumor effects by the ADCC mechanism. Fragment C of the mAb binds to the Fc receptors (FcR) carried by immune cells, thus triggering tumor cell lysis. Functional polymorphisms on two FcR genes (*FCGR2A, FCGR3A*) affecting the affinity of FcR for fragment C have been identified [37,38]. These polymorphisms may thus influence ADCC efficiency [39,40]. Even though some studies have reported significant associations between these polymorphisms and clinical efficacy of rituximab [41], trastuzumab [42] or cetuximab [43,44], data conflict regarding which alleles are linked to favorable patient outcome. In the present study, we report a significant influence of *FCGR3A *F158V polymorphism on survival both in the whole population and in patients with a wt KRas tumor, with VV patients presenting a dramatically shorter survival. The favorable influence of the *FCGR3A *158F allele was also reported in a study by Zhang et al. [43] and a study by Pander et al. [45], but not in the study from Bibeau et al. [44]. These discrepancies related to the impact of *FCGR3A *158F/V polymorphism on cetuximab efficacy are difficult to account for but could be due to the relatively limited sample size of these studies.

We observed that none of the 13 patients pre-treated with bevacizumab had a response to cetuximab and that this subgroup had a significantly decreased specific survival as compared with non-pretreated patients (9.8 months *vs *4.9 months, p = 0.018). This difference remains statistically significant in a multivariate analysis adjusted for age, sex, PS status and KRas status (data not shown). This negative influence of bevacizumab pre-treatment cannot be imputed to patient characteristics which were not significantly different between bevacizumab pretreated patients and non-pretreated patients, although it must be noted that 42% of bevacizumab pretreated patients carried *KRAS *mutated tumors *vs *29% in non-pretreated patients (p = 0.48). Importantly, a multivariate analysis including bevacizumab pretreatment revealed that *CCND1 *A870G and *FCGR3A *F158V polymorphisms both remained significant independent predictors of patient survival (whole population and KRas wt tumors).

The retrospective design of this study, conducted on a relatively small number of patients, may place intrinsic limitations on the present original data. However, results obtained in the sub-group of wt KRas patients, corresponding to the current cetuximab-treated population, clearly suggest that *CCND1 *A870G polymorphism may be used as an additional marker for predicting cetuximab efficacy, TTP and overall survival. Of note, *FCGR3A *F158V polymorphism and *CCND1 *A870G polymorphism were significant independent predictors of overall survival in patients with wt KRas tumors. Such promising observations deserve further confirmation in a prospective study conducted on a larger population of CRC patients receiving cetuximab-based therapy.

## Conflict of interests

we declare that there is no conflict of interest that could be perceived as prejudicing the impartiality of the research reported.

## Authors' contributions

Manuscript writing: LD, GM and MCEG

Review: LD, MCEG, GM

Investigators: LD, EN, JFS, MG

All authors read and approved the final manuscript

## Pre-publication history

The pre-publication history for this paper can be accessed here:

http://www.biomedcentral.com/1471-2407/11/496/prepub
